# Results and generalizability of the Target Temperature Management Trial and future research for patients admitted to intensive care after cardiac arrest

**DOI:** 10.1186/1471-227X-15-S1-A3

**Published:** 2015-06-24

**Authors:** Niklas Nielsen

**Affiliations:** 1 Department of Anesthesiology and Intensive Care, Helsingborg Hospital, Helsingborg, Sweden; Department of Clinical Sciences, Lund University, Lund, Sweden

## 

The Target Temperature Management (TTM) Trial randomized 950 unconscious, adult patients with return of spontaneous circulation after out-of-hospital cardiac arrest (CA) of presumed cardiac cause to strict temperature management at either 33°C or 36°C. Temperature was managed with intravascular or surface cooling devices for 36 hours, while the patients were sedated and mechanically ventilated. Prognostication and decisions on life-sustaining treatments were postponed until 4.5 to 5 days after CA in the general case [1]. There was no difference in the primary outcome: survival until the end of the trial (mortality 50% in the 33°C group and 48% in the 36°C group, hazard ratio 1.06, 95% confidence interval 0.89 to 1.28, *p* = 0.51) or the secondary outcomes: neurological function at 6 months and adverse events [2]. In a substudy with detailed cognitive assessment, the groups were similar [3].

The TTM Trial has been criticized for imbalances between groups, the long time to reaching target temperature, poor temperature control, the short time to basic cardiopulmonary resuscitation and that wide inclusion criteria might have missed subgroups with potential benefit of the lower temperature. Regarding baseline differences, the adjusted analyses moved the point estimate of the intervention in direction benefit for the 36°C group (hazard ratio 1.14, *p* = 0.18) [2]. The time to reach a temperature below 34°C was similar to large registries [4] and faster than the most influential previous randomized trial [5]. Temperature depicted with ±2 standard deviations will visually give an impression of imprecision, compared with studies reporting the interquartile range [5]. Time to basic life support was reported for the subgroups of patients having bystander cardiopulmonary resuscitation only and was identical to previous reports [6]. Subgroup analyses did not show signals in favor of any of the temperatures, but may suggest potential harm of 33°C in circulatory unstable patients [7].

In summary, the results of the TTM Trial are generalizable to the majority of patients admitted to intensive care after a CA of a presumed cardiac cause and temperature management at either 33°C or 36°C after CA should be regarded as evidence-based medicine. Taking the confidence limits of the TTM Trial primary outcome into account, comprising potential clinically significant benefit of both 33°C and 36°C arms, future randomized trials of intensive care treated CA patients must be large and forming international networks will be imperative to move the field forward. The TTM Trial highlights that we still do not have the final answer as to how to best manage temperature after CA and that many questions about efficacy and effectiveness remain open.

## Financial disclosure

NN has received speaker’s reimbursement from C. R. BARD.

## Declaration

This abstract and supplement was proposed, developed and commissioned by BMC Emergency Medicine and was funded by an educational grant from C. R. BARD, NJ, USA. The published abstract was independently prepared by the author. C. R. BARD had no input into the content.
